# P-383. Do Intraoperative Bile Cultures in Pancreaticoduodenectomy Change Antibiotic Management? A Retrospective Observational Study

**DOI:** 10.1093/ofid/ofae631.584

**Published:** 2025-01-29

**Authors:** Elizabeth Ndungu, Ahmad Kofahi, Lara Zraik, Morgan Jackson, Eliza Beal, Lea Monday

**Affiliations:** Wayne State University, detroit, Michigan; Wayne State University School of medicine, Detroit, Michigan; Wayne State University School of medicine, Detroit, Michigan; Wayne State University School of medicine, Detroit, Michigan; Wayne State University School of medicine, Detroit, Michigan; Wayne State University School of medicine, Detroit, Michigan

## Abstract

**Background:**

Biliary stenting increases post-operative surgical site infections (SSIs) following Whipple procedure due to bactibilia. Intraoperative bile culture (IOBC) is performed to guide empiric therapy for SSIs; however, its utility and impact on antibiotic utilization is poorly studied.

**Figure:**
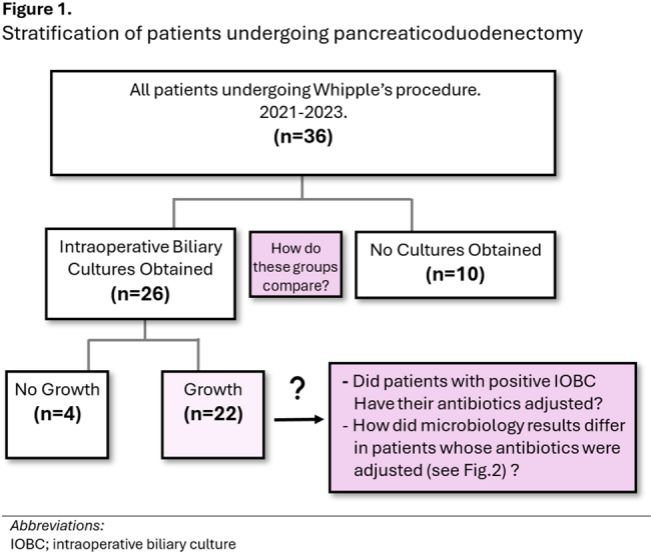

**Methods:**

We performed a retrospective cohort study of adult patients undergoing pancreaticoduodenectomy over a 3 year period (2021-2023). Patients with and without IOBCs were compared for pre and post-operative characteristics and complications. Post-operative antibiotic utilization was also compared between cohorts (Fig1).

**Figure:**
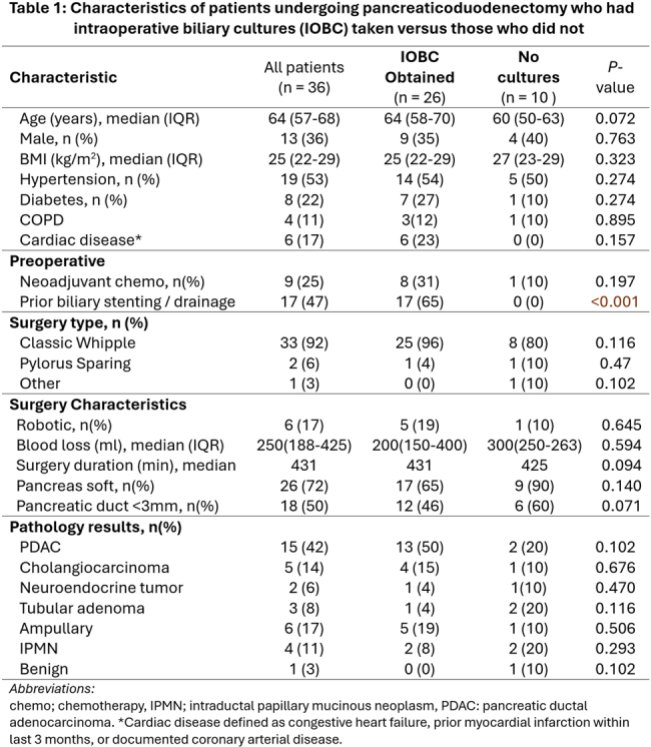

**Results:**

36 patients underwent Whipple’s procedure during the study period; 26 (72%) had IOBC obtained, while 10 (28%) did not. Cohorts were similar in terms of comorbidities, neoplasm, and surgical characteristics (Table 1). All 17 Patients with prior biliary stenting had IOBC obtained, but IOBC were also obtained in 9 patients who had not previously been stented (Table1). Post-operative fever, leukocytosis, SSI, bacteremia, and deep abscess were similar between cohorts, however, more biliary leaks occurred in the group that did not have IOBC (P=0.010)(Table 2a). Compared to the group without cultures, the IOBC group had longer median antibiotic duration (11 versus 3 days, *P*=0.52), and fewer short course antibiotics under 14 days (65% vs 100%, P=0.197), however these were non-significant trends (Table 2b). Patients in the IOBC group also trended toward more antipseudomonal, carbapenem, and antifungal use (P=0.068 to P=0.285, Table 2b). 10 of 22 (45%) of patients with growth on their IOBC did not have antibiotics adjusted based on that result. Patients whose antibiotics were adjusted did not differ in their microbiology results from those whose cultures were ignored (Figure 2).

**Figure:**
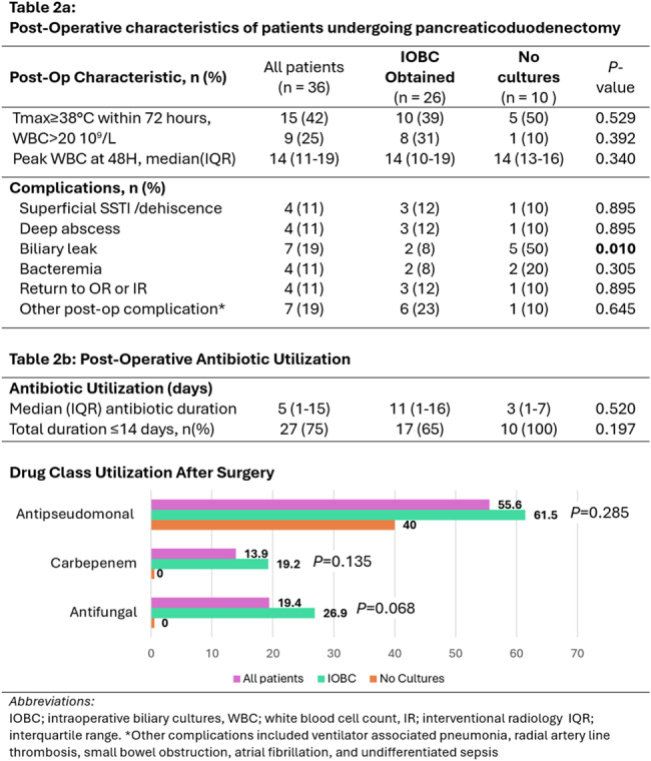

**Conclusion:**

Almost 3 / 4 of patients undergoing Whipple’s procedure had IOBC obtained; some not previously been stented. 45% of patients with positive IOBCs did not have their antibiotics adjusted to target them. Patients who underwent IOBC trended toward longer antibiotic durations and use of broader agents despite having lower rates of post operative biliary leak. These results indicate physicians may feel compelled to treat these cultures without a clinical indication.

**Figure:**
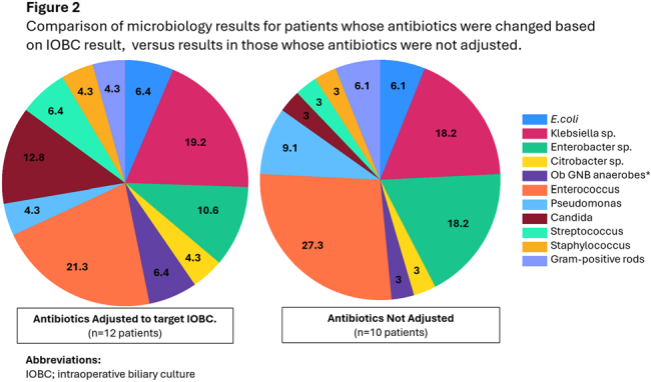

**Disclosures:**

**All Authors**: No reported disclosures

